# Do We Actually Need a New Scale? Improving Postpartum Depression Screening in Malaysia: A Narrative Literature Review

**DOI:** 10.21315/mjms-05-2025-341

**Published:** 2025-08-30

**Authors:** Rania Nafi’ Suleiman Alsabi, Amilia Afzan Mohd Jamil, Rima Anggrena Dasrilsyah, Aishah Siddiqah Alimuddin, Ruthpackiavathy Rajen Durai

**Affiliations:** 1 Department of Nursing, Faculty of Medicine and Health Sciences, Universiti Putra Malaysia, Serdang, Selangor, Malaysia; 2 Department of Obstetrics and Gynaecology, Faculty of Medicine and Health Sciences, Universiti Putra Malaysia, Serdang, Selangor, Malaysia; 3 Department of Psychiatry, Faculty of Medicine and Health Sciences, Universiti Putra Malaysia, Serdang, Selangor, Malaysia

**Keywords:** postpartum depression, screening tools, narrative review, risk assessment, novel scale, Malaysia

## Abstract

Postpartum depression (PPD) is a major maternal health concern in Malaysia, impacting both maternal well-being and infant development. Despite the availability of several validated screening tools, concerns remain about their cultural appropriateness and diagnostic accuracy in Malaysia’s multiethnic context. This narrative review critically evaluates the psychometric properties, contextual relevance, and clinical limitations of PPD screening tools used in Malaysian healthcare. The objective is to assess whether current instruments sufficiently detect PPD in Malaysian women and explore the rationale for a culturally tailored alternative. A targeted literature search was conducted across six databases—PubMed, Scopus, Ovid MEDLINE, Wiley Online Library, Cochrane, and Google Scholar—for studies published between 2000 and 2024, using keywords such as “postpartum depression”, “screening tools”, “Malaysia”, and “psychometric validation”. The inclusion criteria focused on studies examining Malaysian populations and reporting on the implementation, psychometric properties, or contextual adaptation of common screening tools. The Edinburgh Postnatal Depression Scale (EPDS), Postpartum Depression Screening Scale (PDSS), and Patient Health Questionnaire-9 (PHQ-9) were the most commonly used tools. Although they demonstrated moderate validity and reliability, their use in Malaysia is limited by suboptimal linguistic translation, poor contextual adaptation, and inconsistent validation procedures. Furthermore, these tools often overlook key sociocultural determinants like postpartum confinement practices, stigma, and healthcare access disparities. This review highlights the inadequacy of foreign-developed PPD screening tools for Malaysian mothers. Thus, a culturally sensitive, empirically validated tool that incorporates biopsychosocial risk factors specific to Malaysia is warranted to enable timely detection, appropriate intervention, and improved maternal mental health outcomes.

## Introduction

Maternity, often accompanied by extensive preparation for childbirth and newborn care, is widely regarded as a joyful and transformative milestone for women and their families. However, this period can be clouded by postpartum depression (PPD), a serious and prevalent mental health condition affecting mothers and their infants. Commonly, PPD emerges within the first year after delivery, with the majority of cases manifesting during the initial 12 weeks postpartum (1, 2). Moreover, it is recognised as a distinct subtype of major depressive disorder, exhibiting a spectrum of clinical severity and symptom profiles (2). Globally, PPD affects approximately 17.2% of women, has notable regional variations, and the highest prevalence, nearly 40.0%, is observed in Southern Africa (3). In Malaysia, based on the National Health and Morbidity Survey, the prevalence of PPD is 10.4% (4). Despite its substantial burden, many cases remain undetected and untreated, often due to limited mental health awareness, stigma, and barriers to timely screening and intervention (5).

Effective screening tools are essential for the early identification and intervention of PPD. However, their diagnostic utility may be compromised in culturally diverse and multiethnic societies like Malaysia (6). Globally, the Edinburgh Postnatal Depression Scale (EPDS) is one of the most widely adopted instruments. Yet, it predominantly assesses emotional symptoms of depression and often neglects broader biopsychosocial risk factors such as family dynamics, traditional postpartum practices, and social support systems, which are especially relevant in non-Western settings (7). Furthermore, variability in sensitivity and specificity across different cultural contexts introduces a heightened risk of false positives and negatives, undermining diagnostic precision (8, 9). Linguistic limitations, especially those arising from direct translations, can obscure the intended meanings of screening items, leading to misinterpretation among respondents (8). Cultural stigma and prevailing beliefs about mental illness further complicate disclosure, potentially deterring mothers from engaging in self-report screening or seeking care (10).

To address the diagnostic complexities of PPD, several psychometrically validated instruments have been developed. The most recognised and essential tools for early identification and intervention are the Postpartum Depression Screening Scale (PDSS), EPDS, and Patient Health Questionnaire-9 (PHQ-9) (11–13). These instruments have contributed to timely treatment initiation and improved maternal–infant health trajectories. However, in Malaysia’s culturally diverse setting, the utility of these instruments is often limited by differences in symptom expression and cultural interpretations of psychological distress. For instance, Chinese and Indian mothers more frequently report affective symptoms, such as persistent sadness or anxiety, whereas Malay women may present a broader symptom spectrum encompassing emotional, behavioural, and somatic complaints (14). Despite these cultural nuances, healthcare providers often emphasise observable physiological and behavioural signs, potentially overlooking subjective emotional symptoms communicated differently across ethnic groups (10). Furthermore, the predominance of Western-developed tools that lack contextual sensitivity worsens diagnostic gaps in local maternal healthcare pathways (11). Zhao and Zhang (15) emphasised that failure to consider cultural dimensions in screening design contributes to misclassification and underdiagnosis of PPD, especially in low- and middle-income settings. Therefore, there is a pressing need to develop and validate a culturally tailored screening instrument that reflects the biopsychosocial realities of postpartum Malaysian women.

## Methods

This narrative review critically examined the PPD screening tools employed in Malaysia and assessed their cultural relevance, psychometric strength, and diagnostic utility. Unlike systematic reviews, which adhere to rigid inclusion criteria and meta-analytical techniques, this narrative approach facilitates thematic synthesis across diverse study designs. This method is especially useful for addressing context-specific healthcare gaps where localised evidence is limited and heterogeneous.

### Search Strategy

A targeted literature search was conducted across six major databases (PubMed, Scopus, Ovid MEDLINE, Wiley Online Library, Cochrane Library, and Google Scholar), and included articles published between 2000 and 2024. Boolean logic was used to combine search terms such as “postpartum depression”, “PPD”, “screening tools”, “Malaysia”, “psychometric validation”, “EPDS”, “PHQ-9”, “PDSS”, “Beck Depression Inventory”, and “CES-D”. Filters were applied to restrict the results to English-language peer-reviewed articles focusing on Malaysian populations or culturally comparable Southeast Asian cohorts.

### Inclusion Criteria and Study Selection

Studies were selected based on their empirical evaluation of PPD screening instruments. Eligible articles included cross-sectional and cohort studies assessing the reliability, validity, sensitivity, specificity, and cultural appropriateness of the screening tools. Additionally, validation papers that explored cross-cultural adaptation, translation fidelity, and diagnostic performance in diverse maternal populations were included to support broader thematic insights. Opinion pieces, editorials, and conference abstracts were excluded.

## Results

### Screening Protocols and Clinical Guidelines in Malaysia

This narrative review evaluated prevailing PPD screening practices in Malaysia, focusing on clinical protocols, national recommendations, and the most frequently implemented screening instruments. Malaysian healthcare settings typically adopt a two-tiered screening approach for early identification of depressive symptoms among postpartum women.

The initial step often involves the use of brief screening tools like the Patient Health Questionnaire-2 (PHQ-2) and the Whooley Questions. The PHQ-2 is a self-administered Likert-type questionnaire that assesses the frequency of core depressive symptoms (e.g., anhedonia and low mood) over the past 2 weeks. Conversely, the Whooley Questions consist of two binary-response items administered by clinicians to rapidly detect potential depressive symptoms. Both instruments are designed for rapid implementation during routine maternal health follow-up visits.

According to international guidelines, including those outlined by the National Collaborating Centre for Mental Health (16), a positive response on the PHQ-2 or Whooley Questions should prompt further assessment using more comprehensive tools like the EPDS or the PHQ-9. These instruments provide a broader symptom profile and are typically administered during early postnatal consultations in primary healthcare or obstetric settings. Women presenting with moderate to severe symptomatology are generally referred to family medicine or mental health services for formal diagnostic evaluation and follow-up care. However, the implementation of this protocol varies across Malaysian healthcare institutions, with disparities in adherence, tool adaptation, and availability of mental health services contributing to inconsistent screening outcomes.

### Common Tools Used in PPD Screening

In Malaysia, several psychometric instruments are routinely used to screen for PPD, with the EPDS and the PDSS being the most widely implemented tools. These tools have demonstrated favourable psychometric properties, both reporting sensitivity and specificity exceeding 80% (8, 9). The PHQ-9 and the Beck Depression Inventory (BDI) are the other screening instruments commonly referenced in the literature. Although these tools are not developed specifically for postpartum populations, they are often adapted in maternal healthcare settings owing to their accessibility and established clinical utility (9). Despite the widespread use of these tools, their diagnostic accuracy in culturally diverse populations remains variable, necessitating further validation in the Malaysian context.

#### EPDS

Cox et al. (12) developed the EPDS to facilitate the early detection of PPD. This 10-item self-report questionnaire asks women to reflect on their emotional state over the previous 7 days, yielding a total score ranging from 0 to 30. The EPDS is one of the most extensively validated instruments for PPD screening across diverse populations and languages (9, 17). With a suggested cut-off score of 9/10 indicating possible PPD (6), the tool has demonstrated promising diagnostic performance. O’Connor and colleagues (18) reported EPDS sensitivity and specificity values of 0.67–1.00 and 0.87–0.99, respectively, when applied at a threshold of 13 points.

In the Malaysian context, Ruslan et al. (19) evaluated the psychometric robustness of the Malay-translated EPDS based on evidence from local clinical trials conducted over the past decade. Their findings highlighted the EPDS as a flexible and widely used screening instrument. The tool’s reliability was rated as “excellent to exceptional” in Western populations. However, it was rated as “good” in Eastern settings, reflecting possible cultural response variations and the need for further contextual adaptation.

#### PDSS

The PDSS (11) is a specialised self-report instrument developed to identify mothers at risk for PPD. Comprising 35 items, the PDSS offers a comprehensive assessment of the emotional, cognitive, and somatic aspects of PPD, reflecting the multifaceted experiences of women in the postpartum period (20). The PDSS is designed for efficient use in clinical and community settings, typically requiring 5–10 minutes for completion. It uses a scoring range of 0–28, with higher scores indicating increased severity of depressive symptoms. A commonly applied cut-off score of 9 suggests the need for further diagnostic evaluation and appropriate intervention (20). The tool demonstrates excellent diagnostic performance, with sensitivity and specificity values of 91%–94% and 72%–98%, respectively. These metrics reflect its strong capacity to detect true cases of PPD while minimising false positives, making it a valuable asset in maternal mental health screening.

#### PHQ-9

The PHQ-9 (13) is the third most widely utilised screening instrument for PPD; however, it was originally developed for use in the general population. This nine-item self-report tool evaluates core symptoms of depression based on the American Psychiatric Association (21) including mood disturbances, sleep and appetite changes, feelings of guilt or worthlessness, difficulty concentrating, psychomotor changes, and suicidal ideation. Despite not being tailored exclusively for postpartum populations, the PHQ-9 has proven effective in identifying symptoms consistent with major depressive disorder. It demonstrates sensitivity levels ranging from 75% to 90%, supporting its utility in perinatal mental health assessments (22). Its specificity generally falls between 85% and 95%, improving diagnostic accuracy by minimising false-positive results. Moreover, the tool’s brevity, diagnostic alignment, and strong psychometric properties contribute to its continued use in postpartum screening protocols.

#### BDI

The BDI is a widely utilised instrument comprising 21 self-reported items, each rated on a 4-point Likert scale, yielding a total score range of 0–63 (23). Originally developed to measure the severity of depressive symptoms, the BDI has been translated into multiple languages, including Finnish, French, and Korean, to broaden its applicability. However, postpartum women have expressed concerns regarding the tool’s heavy emphasis on somatic symptoms, which may overlap with normal postnatal physiological changes, potentially affecting diagnostic accuracy during the postpartum period (24).

Nevertheless, emerging research suggests that somatic symptoms remain clinically relevant in the context of PPD and should not be overlooked in diagnostic frameworks (25). The BDI has been employed in at least two Malaysian-based studies evaluating its psychometric performance in postpartum populations (26, 27). Reported sensitivity and specificity values ranged between 86% and 88.9% and 78.5% and 88.2%, respectively, with optimal cut-off scores falling between 11 and 14.

#### Centre for Epidemiologic Studies Depression Scale (CES-D)

The CES-D is a 20-item self-report scale designed to assess the frequency of depressive symptoms over the past week, with a total score range of 0–60, indicating increasing levels of depressive severity (28). Studies have validated its effectiveness in identifying PPD, noting that its diagnostic accuracy is comparable to established tools such as the EPDS and PHQ-9 (19, 29). Notably, the CES-D has demonstrated excellent predictive validity for late-life depression in older populations, often performing similarly to the Geriatric Depression Scale (GDS) (30). It has also been applied to postpartum populations to assess depressive symptoms and explore sociodemographic risk factors, including low income and limited educational attainment (31). However, as Abdullah et al. (9) emphasised, different screening tools may yield varying prevalence estimates, reflecting disparities in cut-off thresholds and cultural sensitivity. According to their review, two Malaysian-based studies employed the CES-D for postpartum screening. These studies reported sensitivity and specificity values ranging from 72.7% to 82.6% and 78.5% to 88.2%, respectively, using cut-off scores between 17 and 21 (9, 22).

## Discussion

### Cultural and Clinical Limitations of Current PPD Screening Tools in the Malaysian Setting

In 2019, the Malaysian Ministry of Health (32) released the second version of the Clinical Practice Guidelines for the Management of Major Depressive Disorders, providing up-to-date guidance for medical professionals. These guidelines emphasised that healthcare professionals manage patients based on clinical presentation and the healthcare resources available within their communities. However, no screening tool for PPD has received consensus of use in Malaysia. The existing PPD scales, although valuable in assessing the mental health of new and existing mothers, exhibited several limitations when applied to Malaysian mothers. The critical areas of these limitations are described in the following subsections.

### Cultural and Linguistic Mismatch

Cultural sensitivity and linguistic considerations are essential for effectively screening and diagnosing PPD across diverse populations. Cultural context can significantly influence the expression of PPD symptoms (33). In Malaysia’s multicultural society, comprising Malay, Chinese, Indian, and indigenous groups, emotional distress often presents somatically, that is, through complaints of physical discomfort or fatigue, rather than through explicit expressions of sadness or anxiety (34). This culturally embedded tendency can result in underdiagnosis or misclassification of PPD when screening tools developed in Western contexts are used without appropriate cultural adaptation.

For example, PPD screening tools like the EPDS are widely used in Malaysia but may lack cultural relevance. Translations into Malay, Mandarin, or Tamil often overlook local idioms or nonemotional expressions of distress, e.g., *angin* (wind-related malaise), which are common among Malay women and may result in missed diagnoses (32). A study by Srisurapanont et al. (35) in Thailand found that PHQ-9 demonstrated significantly greater diagnostic accuracy in postpartum women, despite the EPDS and PHQ-9 showing convergent validity with functional disability. This finding suggests that even within the same geographic region, differences in linguistic precision and symptom expression can impact tool performance.

Linguistic diversity within Malaysia adds another layer of complexity to PPD screening. Normal back-to-back translation methods may not fully preserve the semantic distinctions required for diagnostic accuracy (10, 36). Consequently, these tools may fail to detect the full spectrum of depressive symptoms experienced by Malaysian mothers, undermining their reliability and validity (37). When the language of the tool does not align with local expressions, misclassification and missed diagnoses are likely to occur (38).

Thus, validation is essential to ensure that culturally and linguistically adapted tools accurately measure intended constructs in local populations. Key psychometric properties like sensitivity and specificity can be compromised if the adaptation process is inadequate (39). Norhayati et al. (40) emphasised that tools must be rigorously validated in each cultural context to support effective clinical use. Finally, a Vietnamese scoping review by Nguyen et al. (41) revealed that, despite the high rates of PPD (up to 20%), many women avoided professional mental health services, turning instead to informal resources such as fortune-tellers and community beliefs. This hesitancy to seek formal care, which is also mirrored in parts of Malaysia, underscores the importance of culturally aligned screening strategies to ensure timely diagnosis and intervention.

### Limitations of Existing Scales for the Malaysian Population

Globally, PPD screening tools such as the EPDS, BDI, PHQ-9, and DASS-21 are widely utilised. However, applying these tools in culturally different contexts can compromise their effectiveness (42). According to Salehi et al. (6), culturally appropriated versions of these tools made to better suit women from different cultures often relied on low cut-off scores to stay effective because women from different cultures interpret and present depressive symptoms in different ways.

The PPD screening tools need to adapt to different cultural settings because depressive symptoms have varying presentations in each culture. In collectivist societies like Malaysia, emotional distress is often presented through somatic complaints rather than through overt expressions of sadness or hopelessness, which are more prevalent in Western cultures (33). This difference in culture shows how easy it is to misinterpret symptoms when screening tools are not adapted to suit the culture. Additionally, it shows how important it is to ensure that these tools are in line with how women in the community express distress (8).

Although postpartum mothers widely use the EPDS to assess depressive symptoms, its original design did not make it a comprehensive screening tool. The EPDS primarily focuses on depressive symptoms and neglects broader risk factors associated with PPD, e.g., pre-existing mental health disorders, socioeconomic status, marital status, and a history of domestic violence (7). This limitation can result in incomplete assessments of mothers’ mental health, as these factors are significant contributors to the risk of developing PPD.

Research on the EPDS has revealed variability in its sensitivity and specificity across different studies. Reported specificity ranges from 47.7% to 95.6% (8, 9), whereas sensitivity consistently exceeds 80.0%, which indicates a high true positive rate. The inconsistency in specificity can lead to false positives, potentially causing unnecessary anxiety among women who do not have PPD. These differences could be attributed to different ways of validating results in different cultural settings, highlighting the importance of making region-specific changes to the EPDS (9).

Language and interpretation barriers also pose challenges in the effective use of PPD screening tools. Direct translations of these tools might miss culturally specific differences in how women express emotions, which would make them less useful and accurate. To enhance the effectiveness of these tools, it is essential to engage with local experts during the adaptation process, ensuring that language and terminology align with cultural norms (8). Culturally relevant adaptations foster trust in the screening process and enhance the validity of the results.

To make PPD screening tools more culturally relevant, Cardona et al. (43) suggested that researchers should use qualitative methods, involve participants, and use culturally safe ways to conduct the tests. Thus, more research is warranted to make these tools more useful for a wider range of individuals and to make them more reliable and effective for Malaysian women with PPD (44).

### Inconsistent Administration and Interpretation

Beyond cultural limitations, significant gaps exist in the timing, setting, and standardisation of PPD screening practices across Malaysian healthcare institutions. Current screening protocols are typically initiated between four to six weeks postpartum (42), which may be too late to capture early-onset symptoms that emerge within the first days or weeks postpartum (45). This delay reduces the opportunity for timely intervention, potentially allowing symptoms to progress and impact maternal–infant bonding and recovery. Additionally, the accessibility of screening varies significantly across clinical settings. Tools like the EPDS and PHQ-9 are frequently administered in obstetrics and gynaecology clinics but are underutilised in primary healthcare facilities, paediatric clinics, and rural healthcare centres despite being critical contact points for postpartum women (44, 46). Although a national protocol for PPD screening exists, its implementation typically begins after the early postpartum period, often after the first week following delivery, potentially missing cases of early-onset PPD. This timing, combined with variability in implementation across healthcare settings, contributes to inconsistent detection and follow-up outcomes. Moreover, there are notable deficiencies in referral systems and follow-up procedures. Many women who screen positive for depressive symptoms do not receive adequate referral to mental health professionals, owing to a lack of awareness, insufficient service availability, or absence of streamlined healthcare pathways (47, 48). This disconnect weakens the clinical utility of screening and limits opportunities for recovery-oriented intervention. Finally, variability in results is partly attributed to inconsistencies in who administers the screening tools. Some tools, like the Whooley Questions, require trained professionals to sensitively conduct interviews, whereas others, like the EPDS, are often self-administered with minimal guidance. This variation in delivery affects the reliability of interpretation and may contribute to underdiagnosis and misclassification (49, 50). These systemic limitations point to an urgent need for a nationally endorsed, culturally sensitive, and operationally feasible screening strategy that integrates early detection with coordinated referral and support mechanisms across all maternal healthcare settings.

### Stigma and Disclosure Barriers

Mental health stigma remains a significant barrier to effective PPD screening and treatment, particularly in culturally conservative or collectivist societies like Malaysia. Women may hesitate to discuss their emotions or seek treatment owing to cultural expectations that idealise motherhood as a time of joy and emotional stability (38). This societal pressure often fosters feelings of guilt, shame, or inadequacy in mothers experiencing depressive symptoms (51).

Many Malaysian women internalise stigma and avoid disclosing their symptoms for fear of being judged or misunderstood (52, 53). In rural and traditional communities, additional layers of stigma may arise from beliefs associating mental illness with spiritual weakness or dishonour to the family (15, 54). This has direct implications for screening effectiveness. Tools like the EPDS, originally developed in Western settings, may not account for culturally nuanced expressions of distress, resulting in potential underdiagnoses (36).

Furthermore, stigma-induced silence obstructs the utility of social and psychosocial screenings, which rely heavily on candid self-disclosure. Women may suppress information about family tension or financial strain, diminishing the tools’ ability to detect high-risk cases (10). This aligns with findings by Qi et al. (55) and Grinker (56), who emphasised that internalised stigma often leads to isolation, self-blame, and worsening depressive symptoms. However, cross-cultural comparisons reinforce these barriers. In some Asian and Middle Eastern societies, women reportedly conceal symptoms to protect family reputation (57), mirroring trends found in Malaysia. Thus, addressing stigma through culturally responsive public health messaging and the development of stigma-sensitive tools is essential. Moreover, normalising perinatal mental health struggles through education, media engagement, and healthcare provider training could reduce shame, encourage help-seeking, and improve detection and healthcare outcomes.

### Inadequate Consideration of Biopsychosocial Risk Factors

Although PPD screening tools such as the EPDS, PHQ-9, and BDI have demonstrated clinical utility in detecting depressive symptoms, they often fail to capture the complex interplay of biopsychosocial risk factors that contribute to PPD in diverse populations. These tools primarily focus on emotional or cognitive symptoms, neglecting biological influences like hormonal shifts and genetic vulnerability, both of which play a crucial role in postpartum mood disturbances. Women with a history of premenstrual syndrome (PMS) (58) or familial depression (59) are particularly susceptible because of heightened sensitivity to hormonal fluctuations.

Psychologically, a prior history of depression (60) or anxiety (55) significantly increases the risk of developing PPD. Additionally, personality traits such as low self-esteem and poor coping mechanisms, which are often overlooked in standard screening, can influence the severity and course of depressive symptoms (58). Social determinants, including poor marital support, family conflict, or financial stress, remain under-assessed by most screening tools despite exacerbating the risk of developing PPD (58, 60). For example, many working mothers face dual stressors from employment obligations and childcare responsibilities, increasing vulnerability to emotional strain (60).

These omissions suggest that widely used tools may not provide a comprehensive assessment of a mother’s mental health within the Malaysian context. A more inclusive approach that integrates social, economic, psychological, and biological domains is essential for accurate diagnosis and effective care planning. Therefore, developing a contextually adapted, multidimensional screening tool for the Malaysian population is urgently needed to reflect the true complexity of PPD risk.

### Toward a Novel Malaysian Screening Tool for PPD

To address the identified limitations, this review supports the development of a culturally appropriate, risk-based tool specifically tailored to meet Malaysian mothers’ needs. Although screening tools such as the EPDS, PHQ-9, and PDSS demonstrate moderate psychometric properties, their application in Malaysia is constrained by cultural misalignment, varying administration standards, and limited integration into early postnatal healthcare pathways (14, 61). These tools, originally developed in Western populations, primarily focus on symptom detection rather than the assessment of culturally relevant risk factors. Differences in emotional expression, mental health literacy, and help-seeking behaviours among Malaysia’s major ethnic groups, Malay, Chinese, and Indian, can compromise the accuracy and sensitivity of these tools, potentially resulting in misclassification, underreporting, and false positives (14, 61). Moreover, current PPD screening practices in Malaysia often start four to six weeks postpartum, a timeline that risks overlooking early-onset symptoms (42). Delayed detection diminishes treatment opportunities and exacerbates the emotional and psychological burden on new mothers (62). This challenge is compounded by the lack of consensus on when risk begins and ends. As noted by O’Hara and McCabe (63), inconsistencies in measuring PPD obscure prevalence rates and limit clinical interpretation. Similarly, Yin et al. (64) highlighted the importance of evaluating risk factors alongside symptoms to more accurately understand and predict PPD onset.

Thus, a Malaysian-specific tool that emphasises predictive risk factors offers a practical and culturally resonant alternative. Guided by the biopsychosocial model of care by Engel (65), this approach recognised the complex interplay of biological vulnerability, psychological stressors, and social determinants in shaping postpartum mental health. The Postpartum Depression Risk Scoring (PDRS) tool proposed in this review is designed to reflect these factors by incorporating culturally grounded variables such as traditional confinement practices, familial pressure, and informal social support systems. Importantly, the PDRS model offers flexible utility across healthcare settings. It can be administered during routine obstetric visits or opportunistically by paediatricians during well-baby check-ups, expanding the reach of mental health screening without increasing provider burden (68). A short, structured screening format also addresses geographic and time-related constraints that often limit access to mental health services in rural or under-resourced areas (18).

### Potential Community Impact of the PDRS Scale

Malaysians report high satisfaction with public and private healthcare services (67). Studies in Malaysia have shown a notable decline in PPD prevalence between one and six months postnatally. These findings are largely based on the Malay version of the EPDS, with a cutoff score > 12, typically administered two weeks after childbirth to distinguish PPD from transient postpartum blues (47). However, despite growing awareness, no randomised controlled trials in Malaysia have evaluated evidence-based interventions for PPD, and more robust studies are necessary before national-scale implementation can be justified.

The development of a PDRS chart must be guided by key healthcare considerations, including cost-effectiveness, clarity of referral pathways, potential risks of false positives or negatives, and the feasibility of integrating such a tool into existing maternal healthcare services. The PDRS should exhibit strong psychometric properties, e.g., high sensitivity, specificity, simplicity, and interpretability, and should be operationally compatible with urban and rural healthcare systems. Implementing the PDRS offers several advantages over tools such as the EPDS, PHQ-9, and PDSS. The PDRS chart provides a structured, culturally contextualised approach to identifying women at high risk for PPD early in the postnatal period. It may serve as a practical and accessible public health screening solution that can be administered by trained nurses and midwives, rather than mental health professionals, immediately after delivery. Screening tools that incorporate validated risk factors and are tailored to local cultural and healthcare realities can more accurately predict the likelihood of developing PPD (68). Early identification of risk factors is crucial to developing targeted, population-specific interventions aimed at preventing PPD and mitigating its adverse effects on mothers and their infants (43).

Thus, the PDRS chart has the potential to serve as a novel and scalable screening tool. Particularly, it is well-suited to Malaysian mothers and comparable populations in settings with a limited mental health workforce. Critically, it can be administered on day 1 postpartum, unlike existing tools that are commonly used at later timepoints, often between two and six weeks postpartum. Integrating the PDRS tool into postnatal healthcare facilities can facilitate early identification of depressive risk and timely clinical intervention. Table 1 presents a comparative overview of the PDRS chart’s expected advantages in comparison with currently available PPD screening instruments used in Malaysia.

### Limitations of the Proposed PDRS Tool

Despite the proposed advantages of the PDRS chart, its limitations must be acknowledged. First, the development of a standardised, evidence-based risk-scoring system requires comprehensive psychometric validation across diverse subgroups of the Malaysian population. This includes linguistic, ethnic, and socioeconomic representation to ensure applicability and fairness (69). Second, PPD is influenced by a dynamic interplay of biological, psychological, and social factors. These variables often change over time; thus, a static risk-scoring system may not adequately capture individual variability in symptom presentation or risk trajectory (70). Third, although individualised care is ideal, risk factors like cultural beliefs, traditional confinement practices, and informal caregiving norms may not be uniformly represented across all communities (71). Fourth, ethical considerations related to the collection and handling of sensitive psychosocial data must be addressed. Ensuring data privacy, informed consent, and non-stigmatising interpretation are essential for successful integration of the PDRS tool into clinical settings (72). Finally, as knowledge of PPD continues to evolve, future iterations of the scale may require updates to reflect emerging research findings and changes in maternal health practices (73).

### Limitations of the Narrative Review

This narrative review is subject to several methodological limitations. Unlike systematic reviews, it did not employ a preregistered protocol or formal inclusion and exclusion criteria, which may introduce selection bias. The database search strategy, although comprehensive, was limited to English-language publications and may have excluded relevant studies published in other languages or local Malaysian journals not indexed in major databases. Additionally, the narrative synthesis approach relies on thematic interpretation rather than meta-analytic techniques, which limits the ability to quantitatively compare sensitivity, specificity, and validity metrics across studies. Although efforts were made to identify recent and regionally relevant studies, potential gaps may remain in the representation of certain cultural groups, healthcare settings, or unpublished data. Finally, the lack of uniform methodological quality assessment among the included studies limits the strength of the conclusions drawn regarding the psychometric performance of individual tools. Consequently, systematic reviews and meta-analyses are needed to build a stronger empirical foundation for tool adaptation and PPD screening standardisation in Malaysia.

## Conclusion

This review underscores the need for a novel PPD screening tool tailored to the Malaysian context. The proposed tool aims to integrate up-to-date biopsychosocial risk factors identified in local literature, addressing cultural, linguistic, and systemic limitations observed in existing instruments like the EPDS. Although the EPDS is widely utilised, its limited factorial validity and reliance on self-reported symptoms may compromise diagnostic accuracy across diverse cultural populations. These limitations highlight the need for a paradigm shift from symptom-based screening toward a predictive, risk-based approach. A professionally designed, psychometrically robust risk-scoring tool that accounts for the multifactorial aetiology of PPD, comprising biological, psychological, and social aspects, can enable earlier identification and facilitate preventive interventions. By prioritising a biopsychosocial risk assessment model, future research can improve screening precision and support the development of culturally responsive mental health strategies for Malaysian mothers.

## Figures and Tables

**Table 1:** Comparison between the expected advantages of PDRS and the existing screening tools of PPD in Malaysia

	Comparison	PPD existing tools	PDRS
1	Background	Symptomology	Theoretical framework
2	Difficulty of use	Some are difficult (require a professional)	Easy
3	Duration	Ranging from 5 to 30 minutes	5 minutes
4	Function	PPD screening	PPD detection + referral/Rx plan
5	Items construct	PPD symptoms	PPD risk factors
6	Nature of the tool	Scale	Risk-scoring
7	Region	Global	Malaysia-customed
8	Reliability	Varies	Expected 0.85–0.95
9	Reporting	Clinician-rated: Hamilton Rating Scale for Depression (HRSD), Montgomery-Asberg Depression Rating Scale (MADRS) Self-report: EPDS, BDI, CES-D, General Health Questionnaire (GHQ), Hospital Anxiety and Depression Scale (HADS), PDSS	Clinician-rated
10	Settings	Ob/Gyn clinics	Postnatal wards Ob/Gyn clinics Paediatric clinics Primary healthcare centres
11	Timing	4 to 6 weeks postnatally	From day one, postnatally
